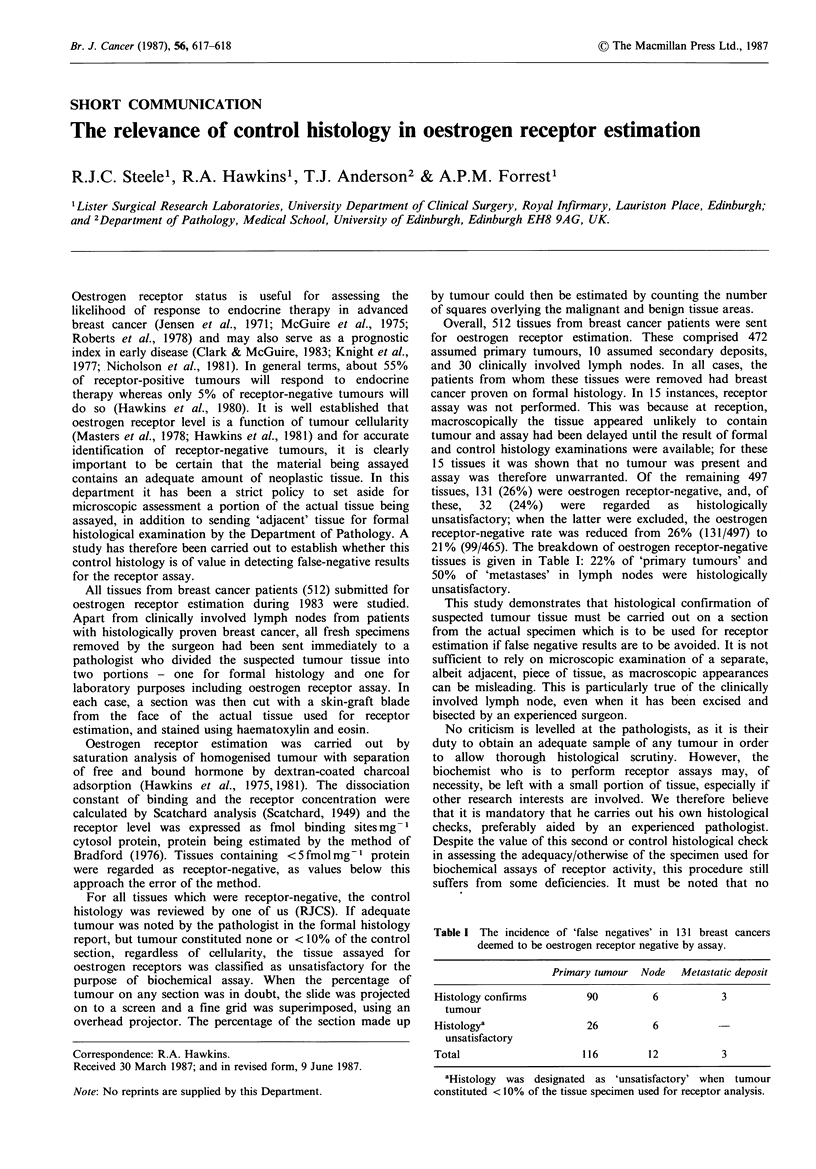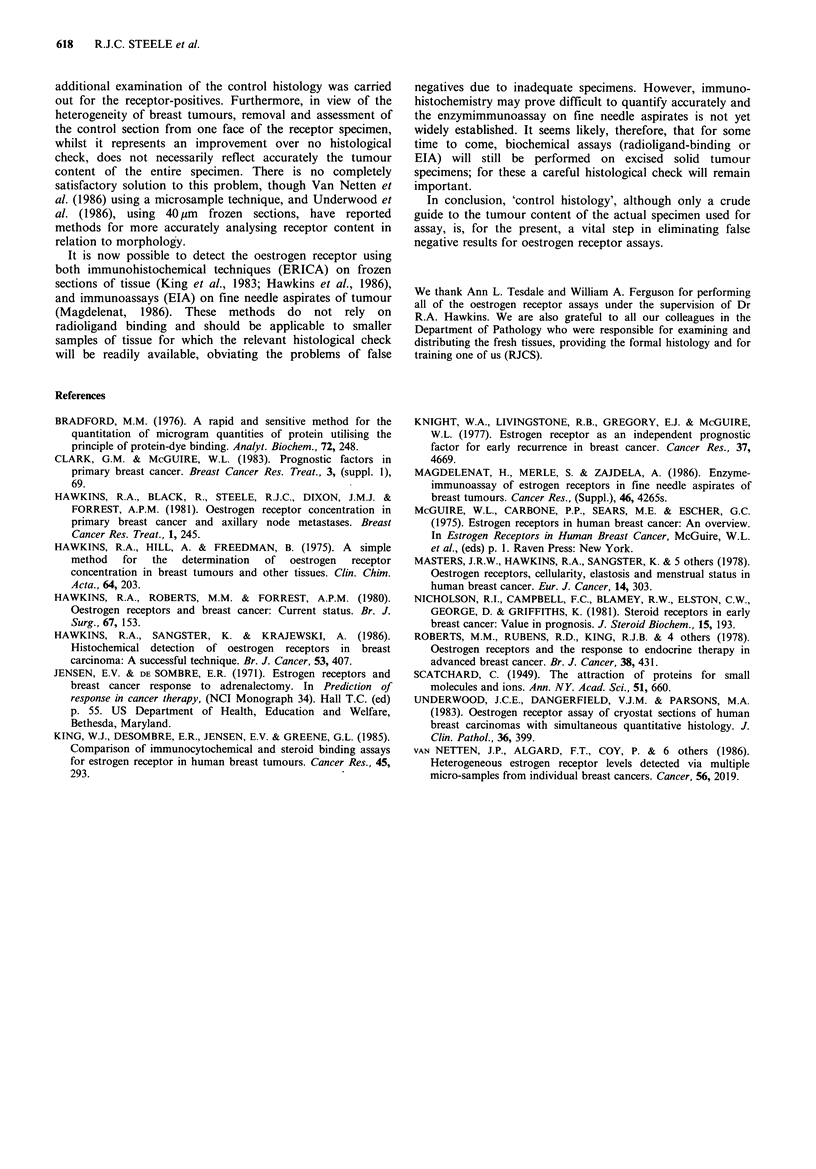# The relevance of control histology in oestrogen receptor estimation.

**DOI:** 10.1038/bjc.1987.252

**Published:** 1987-11

**Authors:** R. J. Steele, R. A. Hawkins, T. J. Anderson, A. P. Forrest

**Affiliations:** Lister Surgical Research Laboratories, University Department of Clinical Surgery, Royal Infirmary, Edinburgh, UK.


Br. J. Cancer (1987), 56, 617–618 © The Macmillan Press Ltd., 1987

SHORT COMMUNICATION

### The relevance of control histology in oestrogen receptor estimation

R.J.C. Steele[1], R.A. Hawkins[1], T.J. Anderson[2] & A.P.M. Forrest[1]

[1]*Lister Surgical Research Laboratories, University Department of Clinical Surgery, Royal Infirmary, Lauriston Place, Edinburgh; and* [2]*Department of Pathology, Medical School, University of Edinburgh, Edinburgh EH8 9AG, UK.*

Oestrogen receptor status is useful for assessing the likelihood of response to endocrine therapy in advanced breast cancer (Jensen *et al.*, 1971; McGuire *et al.*, 1975; Roberts *et al.*, 1978) and may also serve as a prognostic index in early disease (Clark & McGuire, 1983; Knight *et al.*, 1977; Nicholson *et al.*, 1981). In general terms, about 55% of receptor-positive tumours will respond to endocrine therapy whereas only 5% of receptor-negative tumours will do so (Hawkins *et al.*, 1980). It is well established that oestrogen receptor level is a function of tumour cellularity (Masters *et al.*, 1978; Hawkins *et al.*, 1981) and for accurate identification of receptor-negative tumours, it is clearly important to be certain that the material being assayed contains an adequate amount of neoplastic tissue. In this department it has been a strict policy to set aside for microscopic assessment a portion of the actual tissue being assayed, in addition to sending 'adjacent' tissue for formal histological examination by the Department of Pathology. A study has therefore been carried out to establish whether this control histology is of value in detecting false-negative results for the receptor assay.

All tissues from breast cancer patients (512) submitted for oestrogen receptor estimation during 1983 were studied. Apart from clinically involved lymph nodes from patients with histologically proven breast cancer, all fresh specimens removed by the surgeon had been sent immediately to a pathologist who divided the suspected tumour tissue into two portions – one for formal histology and one for laboratory purposes including oestrogen receptor assay. In each case, a section was then cut with a skin-graft blade from the face of the actual tissue used for receptor estimation, and stained using haematoxylin and eosin.

Oestrogen receptor estimation was carried out by saturation analysis of homogenised tumour with separation of free and bound hormone by dextran-coated charcoal adsorption (Hawkins *et al.*, 1975, 1981). The dissociation constant of binding and the receptor concentration were calculated by Scatchard analysis (Scatchard, 1949) and the receptor level was expressed as fmol binding sites $mg^{-1}$ cytosol protein, protein being estimated by the method of Bradford (1976). Tissues containing $<5\,fmol\,mg^{-1}$ protein were regarded as receptor-negative, as values below this approach the error of the method.

For all tissues which were receptor-negative, the control histology was reviewed by one of us (RJCS). If adequate tumour was noted by the pathologist in the formal histology report, but tumour constituted none or $<10\%$ of the control section, regardless of cellularity, the tissue assayed for oestrogen receptors was classified as unsatisfactory for the purpose of biochemical assay. When the percentage of tumour on any section was in doubt, the slide was projected on to a screen and a fine grid was superimposed, using an overhead projector. The percentage of the section made up by tumour could then be estimated by counting the number of squares overlying the malignant and benign tissue areas.

Overall, 512 tissues from breast cancer patients were sent for oestrogen receptor estimation. These comprised 472 assumed primary tumours, 10 assumed secondary deposits, and 30 clinically involved lymph nodes. In all cases, the patients from whom these tissues were removed had breast cancer proven on formal histology. In 15 instances, receptor assay was not performed. This was because at reception, macroscopically the tissue appeared unlikely to contain tumour and assay had been delayed until the result of formal and control histology examinations were available; for these 15 tissues it was shown that no tumour was present and assay was therefore unwarranted. Of the remaining 497 tissues, 131 (26%) were oestrogen receptor-negative, and, of these, 32 (24%) were regarded as histologically unsatisfactory; when the latter were excluded, the oestrogen receptor-negative rate was reduced from 26% (131/497) to 21% (99/465). The breakdown of oestrogen receptor-negative tissues is given in Table I: 22% of 'primary tumours' and 50% of 'metastases' in lymph nodes were histologically unsatisfactory.

This study demonstrates that histological confirmation of suspected tumour tissue must be carried out on a section from the actual specimen which is to be used for receptor estimation if false negative results are to be avoided. It is not sufficient to rely on microscopic examination of a separate, albeit adjacent, piece of tissue, as macroscopic appearances can be misleading. This is particularly true of the clinically involved lymph node, even when it has been excised and bisected by an experienced surgeon.

No criticism is levelled at the pathologists, as it is their duty to obtain an adequate sample of any tumour in order to allow thorough histological scrutiny. However, the biochemist who is to perform receptor assays may, of necessity, be left with a small portion of tissue, especially if other research interests are involved. We therefore believe that it is mandatory that he carries out his own histological checks, preferably aided by an experienced pathologist. Despite the value of this second or control histological check in assessing the adequacy/otherwise of the specimen used for biochemical assays of receptor activity, this procedure still suffers from some deficiencies. It must be noted that no

**Table I** The incidence of 'false negatives' in 131 breast cancers deemed to be oestrogen receptor negative by assay.

| | *Primary tumour* | *Node* | *Metastatic deposit* |
|---|---|---|---|
| Histology confirms tumour | 90 | 6 | 3 |
| Histology[a] unsatisfactory | 26 | 6 | — |
| Total | 116 | 12 | 3 |

[a]Histology was designated as 'unsatisfactory' when tumour constituted $<10\%$ of the tissue specimen used for receptor analysis.

Correspondence: R.A. Hawkins.
Received 30 March 1987; and in revised form, 9 June 1987.

*Note*: No reprints are supplied by this Department.

additional examination of the control histology was carried out for the receptor-positives. Furthermore, in view of the heterogeneity of breast tumours, removal and assessment of the control section from one face of the receptor specimen, whilst it represents an improvement over no histological check, does not necessarily reflect accurately the tumour content of the entire specimen. There is no completely satisfactory solution to this problem, though Van Netten *et al.* (1986) using a microsample technique, and Underwood *et al.* (1986), using 40 $\mu$m frozen sections, have reported methods for more accurately analysing receptor content in relation to morphology.

It is now possible to detect the oestrogen receptor using both immunohistochemical techniques (ERICA) on frozen sections of tissue (King *et al.*, 1983; Hawkins *et al.*, 1986), and immunoassays (EIA) on fine needle aspirates of tumour (Magdelenat, 1986). These methods do not rely on radioligand binding and should be applicable to smaller samples of tissue for which the relevant histological check will be readily available, obviating the problems of false negatives due to inadequate specimens. However, immunohistochemistry may prove difficult to quantify accurately and the enzymimmunoassay on fine needle aspirates is not yet widely established. It seems likely, therefore, that for some time to come, biochemical assays (radioligand-binding or EIA) will still be performed on excised solid tumour specimens; for these a careful histological check will remain important.

In conclusion, 'control histology', although only a crude guide to the tumour content of the actual specimen used for assay, is, for the present, a vital step in eliminating false negative results for oestrogen receptor assays.

We thank Ann L. Tesdale and William A. Ferguson for performing all of the oestrogen receptor assays under the supervision of Dr R.A. Hawkins. We are also grateful to all our colleagues in the Department of Pathology who were responsible for examining and distributing the fresh tissues, providing the formal histology and for training one of us (RJCS).

### References

BRADFORD, M.M. (1976). A rapid and sensitive method for the quantitation of microgram quantities of protein utilising the principle of protein-dye binding. *Analyt. Biochem.*, **72**, 248.

CLARK, G.M. & McGUIRE, W.L. (1983). Prognostic factors in primary breast cancer. *Breast Cancer Res. Treat.*, **3**, (suppl. 1), 69.

HAWKINS, R.A., BLACK, R., STEELE, R.J.C., DIXON, J.M.J. & FORREST, A.P.M. (1981). Oestrogen receptor concentration in primary breast cancer and axillary node metastases. *Breast Cancer Res. Treat.*, **1**, 245.

HAWKINS, R.A., HILL, A. & FREEDMAN, B. (1975). A simple method for the determination of oestrogen receptor concentration in breast tumours and other tissues. *Clin. Chim. Acta.*, **64**, 203.

HAWKINS, R.A., ROBERTS, M.M. & FORREST, A.P.M. (1980). Oestrogen receptors and breast cancer: Current status. *Br. J. Surg.*, **67**, 153.

HAWKINS, R.A., SANGSTER, K. & KRAJEWSKI, A. (1986). Histochemical detection of oestrogen receptors in breast carcinoma: A successful technique. *Br. J. Cancer*, **53**, 407.

JENSEN, E.V. & DE SOMBRE, E.R. (1971). Estrogen receptors and breast cancer response to adrenalectomy. In *Prediction of response in cancer therapy*, (NCI Monograph 34). Hall T.C. (ed) p. 55. US Department of Health, Education and Welfare, Bethesda, Maryland.

KING, W.J., DESOMBRE, E.R., JENSEN, E.V. & GREENE, G.L. (1985). Comparison of immunocytochemical and steroid binding assays for estrogen receptor in human breast tumours. *Cancer Res.*, **45**, 293.

KNIGHT, W.A., LIVINGSTONE, R.B., GREGORY, E.J. & McGUIRE, W.L. (1977). Estrogen receptor as an independent prognostic factor for early recurrence in breast cancer. *Cancer Res.*, **37**, 4669.

MAGDELENAT, H., MERLE, S. & ZAJDELA, A. (1986). Enzyme-immunoassay of estrogen receptors in fine needle aspirates of breast tumours. *Cancer Res.*, (Suppl.), **46**, 4265s.

McGUIRE, W.L., CARBONE, P.P., SEARS, M.E. & ESCHER, G.C. (1975). Estrogen receptors in human breast cancer: An overview. In *Estrogen Receptors in Human Breast Cancer*, McGuire, W.L. *et al.*, (eds) p. 1. Raven Press: New York.

MASTERS, J.R.W., HAWKINS, R.A., SANGSTER, K. & 5 others (1978). Oestrogen receptors, cellularity, elastosis and menstrual status in human breast cancer. *Eur. J. Cancer*, **14**, 303.

NICHOLSON, R.I., CAMPBELL, F.C., BLAMEY, R.W., ELSTON, C.W., GEORGE, D. & GRIFFITHS, K. (1981). Steroid receptors in early breast cancer: Value in prognosis. *J. Steroid Biochem.*, **15**, 193.

ROBERTS, M.M., RUBENS, R.D., KING, R.J.B. & 4 others (1978). Oestrogen receptors and the response to endocrine therapy in advanced breast cancer. *Br. J. Cancer*, **38**, 431.

SCATCHARD, C. (1949). The attraction of proteins for small molecules and ions. *Ann. NY. Acad. Sci.*, **51**, 660.

UNDERWOOD, J.C.E., DANGERFIELD, V.J.M. & PARSONS, M.A. (1983). Oestrogen receptor assay of cryostat sections of human breast carcinomas with simultaneous quantitative histology. *J. Clin. Pathol.*, **36**, 399.

VAN NETTEN, J.P., ALGARD, F.T., COY, P. & 6 others (1986). Heterogeneous estrogen receptor levels detected via multiple micro-samples from individual breast cancers. *Cancer*, **56**, 2019.